# Aiding 30,000 Blind

**Published:** 1920-03-20

**Authors:** 


### Aiding 30,000 Blind.

The House of Commons agreed without dissent to the second reading of a Bill introduced by the Labour Party to provide for the technical education of the blind by the establishment of technical schools where necessary, or by contributions to existing schools and institutions for the employment of the blind, by the establishment of workshops where necessary, or by contributions to existing institutions providing work for the blind. It further aims to provide for grants to augment the wages earned by persons so employed; for the provision of the expenses of blind persons at institutions or hostels while under technical instruction; for the employment and maintenance of blind persons away from workshops; and for the maintenance of blind persons incapacitated from earning their livelihood.

Dr. Addison promised to adopt the suggestion for a more methodical inquiry into the causes and prevention of blindness. He said that the Government proposed to ask for power to require the registration of agencies seeking support of a voluntary character for the blind. He was prepared to support proposals whereby county and borough councils, and possibly other bodies, might be authorised to contribute towards the provision and maintenance of workshops, hostels, etc. The Government proposed that any blind person between the age of fifty and seventy should, subject to the same disqualification as to income and so forth, receive the benefit and allowances granted to old-age pensioners. He suggested that after the second reading he should consult those interested, and then either reform the bill or introduce another in order to give effect to the scheme.

This should give satisfaction to all friends of the blind —there are about 30,000 blind in the United Kingdom, including about 1,500 soldiers—and not the least important part of Dr. Addison's statement is the promise of more methodical inquiry into the causes of blindness. Mr. Tillett, who introduced the Bill, said 20 per cent. of the blind lose their sight within six months of birth, and 25 per cent. of the cases are due to gonorrhea in the mother. Blindness is largely a preventable disease.

